# The frequency of co-occurrence of Hashimoto disease in granulomatous mastitis patients and its relation to hematological parameters

**DOI:** 10.1097/MD.0000000000048758

**Published:** 2026-05-15

**Authors:** Buket Altun Özdemir, Servet Kocaöz, Ömer Yazicioğlu, Bülent Çomçali, Furkan Savaş, Onur Karaca, Aysel Çolak, Birol Korukluoğlu

**Affiliations:** a Department of General Surgery, Ankara City Hospital, Bilkent, Ankara, Turkey; b Department of Medical Pathology, Ankara City Hospital, Bilkent, Ankara, Turkey; c Department of General Surgery, University of Health Sciences, Üsküdar, Istanbul, Turkey.

**Keywords:** C-reactive protein, Hashimoto thyroiditis, Idiopathic granulomatous mastitis, lymphocyte-to-monocyte ratio, neutrophil-to-lymphocyte ratio

## Abstract

**Background:**

Idiopathic granulomatous mastitis (IGM) is a benign, chronic inflammatory breast disease frequently associated with autoimmune disorders. This prospective cohort study explored the prevalence of Hashimoto thyroiditis (HT) in patients with IGM and the relationship between this co-occurrence and systemic inflammatory markers.

**Methods:**

Between March 2019 and September 2022, 179 women were enrolled: IGM without HT (n = 52), IGM + HT (n = 19), and age-matched healthy controls (n = 89). Hematological indices, including white blood cell count, C-reactive protein, neutrophil-to-lymphocyte ratio (NLR), and lymphocyte-to-monocyte ratio (LMR), were measured at diagnosis before any treatment.

**Results:**

The prevalence of HT among patients with IGM was 26.8 %. Compared with isolated IGM, the IGM + HT group had a lower smoking rate (16.3% vs 40.4%, *P* = .028) and displayed a significantly lower median NLR (1.91 vs 2.76, *P* < .001) but higher median LMR (6.47 vs 4.75, *P* = .001).

**Conclusion:**

Approximately 1 in 4 women with IGM also had HT. A low NLR-high LMR pattern may reflect a distinct inflammatory milieu in this overlap cohort and could help tailor follow-up strategies for these patients. Routine thyroid screening should be considered in patients with IGM.

## 1. Introduction

Benign and malignant breast diseases have been frequently observed in patients diagnosed with Hashimoto thyroiditis (HT) and nodular goiter.^[1–3]^ Granulomatous mastitis (GM) is recognized as an autoimmune inflammatory disease characterized by chronic granulomatous inflammation of the breast.^[4–7]^ Clinically, GM may present asymptomatically or manifest through breast nodularity, recurrent episodes of mastitis, nipple discharge, nipple retraction, or regional lymphadenopathy.^[7]^ Among GM subtypes, tuberculous mastitis represents the most prevalent form; hence, it is crucial to differentiate it from tuberculosis and breast carcinoma during diagnosis.^[8]^ Furthermore, granulomatous disorders such as Crohn disease, granulomatous polyangiitis (Wegener granulomatosis), sarcoidosis, autoimmune disorders including Immunoglobin G4-related diseases, and systemic lupus erythematosus must be considered during the clinical assessment of GM.^[9,10]^ Crohn disease, although uncommon, classically causes mastitis presenting as breast masses mimicking abscesses and is histologically characterized by granulomatous inflammation with eosinophilic infiltration.^[11]^ The coexistence of HT and GM as secondary autoimmune diseases has been reported in several studies.^[7–12]^ Despite these associations, no study has specifically explored the prevalence of HT in patients with GM. It has been shown that the neutrophil-to-lymphocyte ratio (NLR) is statistically higher in HT cases than in controls. The NLR, a systemic inflammatory biomarker, can be used for the diagnosis and follow-up of HT.^[13]^ Given these inflammatory and autoimmune overlaps, this prospective study aimed to investigate the prevalence of HT in patients diagnosed with idiopathic GM (IGM). Additionally, we evaluated the impact of HT on prognosis and its relationship with systemic inflammatory markers, including the NLR, lymphocyte-to-monocyte ratio (LMR), platelet-to-lymphocyte ratio (PLR), and C-reactive protein (CRP), in patients with GM.

## 2. Materials and methods

This study is a prospective cohort study conducted from March 1,2019 to September 30,2022 at the Breast and Endocrine Surgery Outpatient Clinic of Ankara City Hospital. Approval was obtained from the Ethics Committee for Clinical Trials of our hospital (E1-20-1415). The inclusion criteria were patients aged > 18 years who were newly diagnosed with GM during the study period. The exclusion criteria were as follows: patients diagnosed before the study date, those whose pathological examinations were performed in other centers, and patients diagnosed with tuberculous mastitis or inflammatory breast cancer. Patients were informed about the study by physicians at the outpatient clinic, and informed consent was obtained from all the participants. One patient with inflammatory breast cancer, 4 with tuberculosis, and 6 who did not wish to participate in the study were excluded. Socio-demographic information, smoking history, family history of granulomatous or autoimmune diseases, and clinical course data were systematically collected. Hematological parameters, including NLR, LMR, PLR, and CRP, were measured at diagnosis before the initiation of any treatment. Whole blood analysis, anti-thyroid peroxidase (TPO), and anti-thyroglobulin (TG) tests were performed on all patients. Thyroid ultrasound was performed exclusively in patients whose anti-TPO or anti-TG levels exceeded the normal laboratory values. The control group was consecutively selected and age-matched, consisting of patients visiting the clinic for benign breast-related concerns without GM or autoimmune thyroid disease.

### 2.1. Data analysis

Data analyses were performed using the SPSS software (version 25.0; IBM Corp.). Numbers, percentages, means, and standard deviations were used to present the descriptive statistical analyses. When the age distribution of patients with and without HT and IGM was analyzed using the Shapiro–Wilk test, the data did not show a normal distribution (*P* < .001). Similarly, when haematological rates in patients with and without HT and IGM were analyzed using the Shapiro–Wilk test, the data did not show a normal distribution (*P* < .001). The significance of the difference between haematological rates showing a systemic inflammatory response in patients with and without HT with IGM was analyzed using the Mann–Whitney U test. The significance of the difference in smoking data between patients with and without HT was analyzed using a continuous correction test. The significance of the differences between groups was tested using the Kruskal–Wallis test for haematological parameters in the 3 groups.

## 3. Results

**Figure 1.**
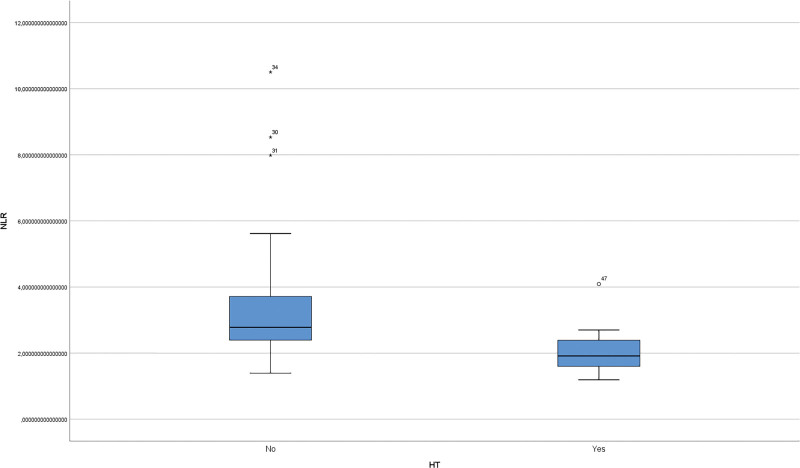
Graph showing the NLR in the blood count of patients with IGM with and without HT. NLR = neutrophil-to-lymphocyte ratio, HT = Hashimoto thyroiditis, IGM = idiopathic granulomatous mastitis.

**Figure 2.**
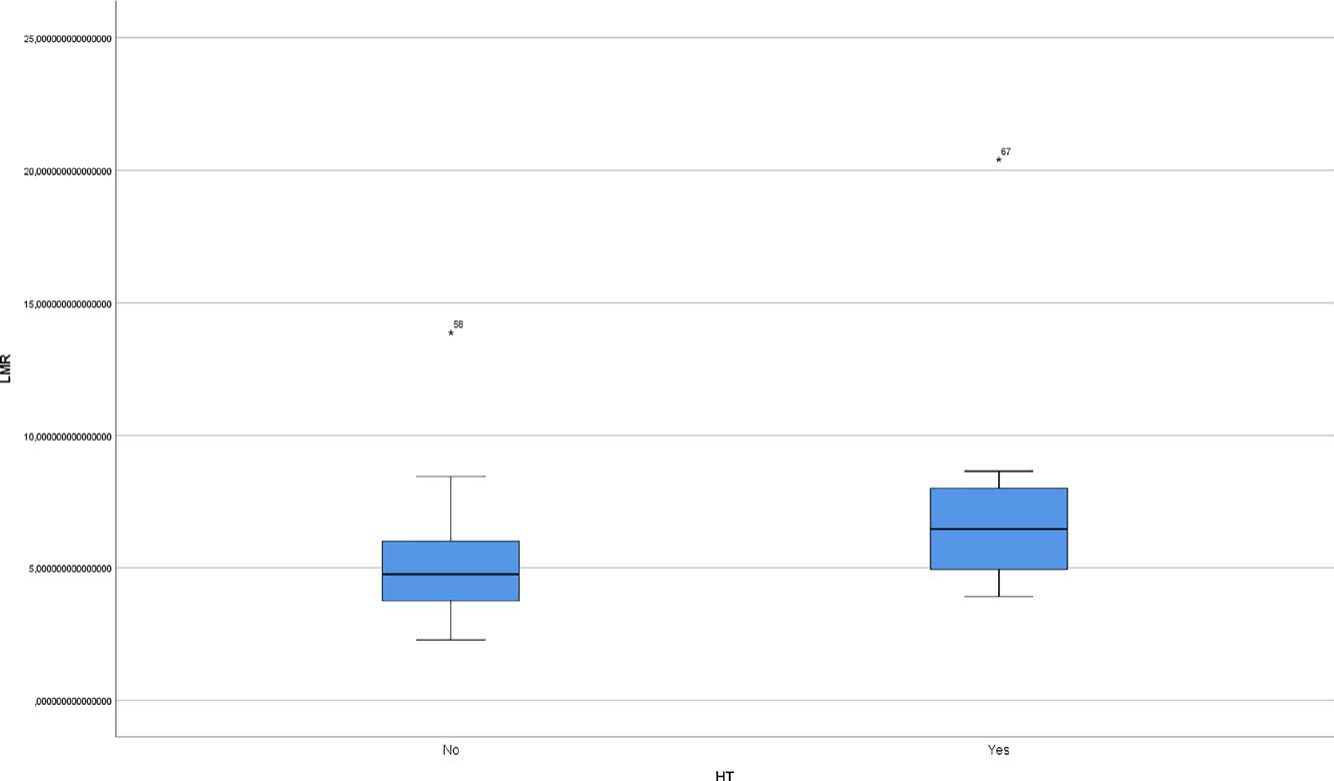
Graph showing the LMR in the blood count of patients with IGM with and without HT. LMR = lymphocyte-monocyte ratio, HT = Hashimoto thyroiditis, IGM = idiopathic granulomatous mastitis.

The median age of the patients in the study was 34 years (min: 22–max: 71 years). Smoking was present in 39.4% (n = 28) of patients with IGM. HT occurred in 26.8% of patients with IGM. Among patients with IGM, the median age of patients without HT was 35 years (min: 22, interquartile range [IQR]: 10, max: 71), and the median age of patients with HT was 33 years (min: 23, IQR: 12, max: 59). Among patients with IGM, the median serum anti-TPO level in patients without HT was 28 U/mL (min: 20, IQR: 4, max: 48), and the median anti-TPO level in patients with HT was 253 U/mL (min: 132, IQR: 979, max: 13,000). Among patients with IGM, the median serum anti-TG level in patients without HT was 0.1 IU/mL (min: 0.1, IQR: 0.1, max: 0.4), and the median anti-TG level in patients with HT disease was 1.8 IU/mL (min: 0.6, IQR: 4.39, max: 27.1). Smoking status was significantly lower in patients with IGM and HT (*P* = .028) (Table 1). The median NLR was 1.91 (min: 1.19, IQR: 0.85, max: 4.09), in patients without HT and 2.76 (min: 1.39, IQR: 1.24, max: 10.5) in patients with coexistence (Fig. 1). The NLR was significantly lower in patients with IGM and concomitant HT (*P* < .001). The median LMR in patients without HT was 4.75 (min: 2.28, IQR: 2.26, max: 13.88), and the median LMR in patients with coexistence was 6.47 (min: 3.91, IQR: 3.38, max: 20.4) (Fig. 2). The LMR was significantly higher in patients with IGM and concomitant HT (*P* = .001) (Table 2). Although the percentage of lymphocytes in IGM patients with HT was low, the difference was not statistically significant (*P* = .225). When only the IGM and IGM + HT groups were compared with the control group, NLR, WBC and CRP levels were significantly higher than in the control group (all *P* < .001). LMR was significantly lower in the IGM group (*P* = .001). A significant decrease in hemoglobin was observed in the IGM + HT group (*P* = .011) (Table 3). Disease recurrence was observed in 19 patients with IGM and in 3 patients with HT (*P* = .128). Although recurrence was more frequent in smokers, the difference was not statistically significant (*P* = .075).

**Table 1:** Comparison of smoking in patients with and without HT with IGM.

			IGM HT association		*P* value
			No	Yes	
Smoking	No	N	16	12	0.028
		%	57.1	42.9	
	Yes	N	36	7	
		%	83.7	16.3	
Total		N	52	19	
		%	73.2	26.8	

HT = Hashimoto thyroiditis, IGM = idiopathic granulomatous mastitis, N = number of patients.

**Table 2:** Comparison of rates showing systemic inflammatory response in patients with IGM with and without HT.

Hematological rates	IGM HT association	N	Median	IQR	*P* value
NLR	No	52	2.74	1.24	< .001
	Yes	19	1.98	0.84	
LMR	No	52	4.65	2.28	.001
	Yes	19	6.45	3.22	
PLR	No	52	142	67.77	.399
	Yes	19	145.63	53.56	
WBC	No	52	9.67	2.94	.018
	Yes	19	7.92	2.94	
CRP	No	52	10.00	15.98	.116
	Yes	19	4.20	10.40	
Hb	No	52	12.80	1.88	.084
	Yes	19	12.10	1.60	

CRP = C-reactive protein, Hb = hemoglobin, HT = Hashimoto thyroiditis, IGM = idiopathic granulomatous mastitis, IQR = interquartile range, LMR = lymphocyte-to-monocyte ratio, N = number of patients, NLR = neutrophil-to-lymphocyte ratio, PLR = platalet-to-lymphocyte ratio, WBC = white blood count.

**Table 3:** Comparison of the rates of systemic inflammatory response in IGM patients with and without HT with the control group.

Hematological rates	IGM HT association	N	Median	IQR	*P* Value
NLR	IGM	52	2.74	1.22	< .001
	IGM + HT	19	2.04	0.85	
	Control	89	2.00	0.87	
LMR	IGM	52	4.75	2.38	.001
	IGM + HT	19	6.43	3.38	
	Control	89	5.74	1.95	
PLR	IGM	52	299.50	106.75	.061
	IGM + HT	19	263.00	72.00	
	Control	89	248.00	71.00	
WBC	IGM	52	9.65	2.91	< .001
	IGM + HT	19	7.75	3.06	
	Control	89	6.75	2.08	
CRP	IGM	52	10.00	15.98	< .001
	IGM + HT	19	4.20	10.40	
	Control	89	4.00	3.80	
Hb	IGM	52	12.80	1.88	.011
	IGM + HT	19	12.10	1.60	
	Control	89	13	1.65	

CRP = C-reactive protein, Hb = hemoglobin, HT = Hashimoto thyroiditis, IGM = idiopathic granulomatous mastitis, IQR = interquartile range, LMR = lymphocyte-to-monocyte ratio, N = number of patients, NLR = neutrophil-to-lymphocyte ratio, PLR = platalet-to-lymphocyte ratio, WBC = white blood count.

## 4. Discussion

Smoking is considered one of an etiological factor in IGM. However, the exact association between smoking and IGM remains to be elucidated. Baslaim et al reported no history of smoking among their IGM patients,^[14]^ while other studies have noted varying smoking prevalence ranging from 16.7 to 77.8%.^[15,16]^ Additionally, smoking has been implicated in increased recurrence rates (6–12%).^[17,18]^ Previous studies have also demonstrated that smoking negatively affects thyroid function, influencing iodine uptake and thyroid-stimulating hormone levels,^[19,20]^ exacerbating ophthalmopathy in Grave disease, and increasing relapse rates.^[19,21]^ In our study, smoking prevalence was significantly lower in the group with concurrent IGM and HT compared to those with IGM alone, though the increased recurrence rate in smokers was not statistically significant. Autoimmune thyroid diseases have been associated with lymphocytic mastitis, as evidenced by elevated anti-TPO and anti-TG levels.^[12,22]^ To our knowledge, this study uniquely investigated the prevalence of coexisting HT in patients with IGM, reporting a significantly higher prevalence (26.8%) than that in the general population, which has been reported to range between 5% and 10% in both smaller cohorts and large-scale population-based studies.^[23,24]^ This finding underscores the importance of screening for HT in patients with IGM, as timely detection can facilitate targeted therapeutic interventions and potentially improve patient outcomes.

Several studies have highlighted the clinical utility of NLR as an inflammatory biomarker in HT, consistently reporting elevated NLR levels compared to healthy controls.^[25–27]^ CRP, NLR, and PLR have also emerged as potential nonspecific indicators of immune dysregulation in autoimmune diseases, although their precise roles remain unclear.^[28,29]^ In the context of IGM, elevated preoperative NLR values have been linked to disease recurrence.^[30–33]^ However, our study observed a lower NLR and higher LMR in patients with IGM and concurrent HT, suggesting a possible unique inflammatory response in this subgroup. Nevertheless, these results should be interpreted cautiously and considered hypothesis-generating, given the potential confounders such as treatment status and timing of blood sampling.

Hematological markers, such as LMR, have demonstrated prognostic value across various diseases, including autoimmune disorders and malignancies, with lower values generally associated with poorer outcomes.^[34,35]^ Although our study identified significant differences in hematological markers, further research is needed to clarify their pathophysiological implications and clinical utility in autoimmune overlap syndromes.

Clinically, these findings highlight the potential benefits of routine thyroid screening in patients diagnosed with IGM, considering the high prevalence of coexisting HT. Early recognition of concurrent thyroid diseases may lead to improved management strategies and outcomes.

One of the limitations of this study is that it was conducted on patients with IGM who were followed up and treated within a certain period. The advantages of the study are that it consists of patients whose medical records are kept regularly and who are followed up personally by the doctors involved in the study

## 5. Conclusion

This prospective study demonstrated a notably higher prevalence of HT among patients with IGM than in the general population. Patients with coexisting IGM and HT exhibited distinct hematological profiles, including lower NLRs and higher LMRs, suggesting a potentially different immunological response pattern. Although smoking appeared less common in the IGM + HT group and disease recurrence was lower, these associations did not reach statistical significance and warrant further investigation in future studies.

From a clinical standpoint, our findings support the potential benefits of routine thyroid screening in patients diagnosed with IGM. Early detection of coexisting thyroid autoimmunity may contribute to more personalized follow-up strategies and improved monitoring of the disease. However, it is important to note that, owing to the observational nature of this study, causal relationships cannot be established. Future multicenter, prospective, and mechanistic studies are needed to confirm these findings and to better understand the immunopathogenic overlap between IGM and autoimmune thyroid disease.

## Author contributions

**Data curation:** Buket Altun Özdemir, Servet Kocaöz, Ömer Yazicioğlu, Bülent Çomçali, Aysel Çolak.

**Formal analysis:** Buket Altun Özdemir, Servet Kocaöz.

**Investigation:** Servet Kocaöz.

**Methodology:** Servet Kocaöz, Aysel Çolak.

**Supervision:** Onur Karaca, Birol Korukluoğlu.

**Visualization:** Bülent Çomçali.

**Writing – original draft:** Buket Altun Özdemir, Servet Kocaöz, Ömer Yazicioğlu, Bülent Çomçali, Aysel Çolak.

**Writing – review & editing:** Furkan Savaş, Onur Karaca, Birol Korukluoğlu.

## References

[1] Anil C Guney T Gursoy A. The prevalence of benign breast diseases in patients with nodular goiter and Hashimoto’s thyroiditis. J Endocrinol Invest. 2015;38:971–5. 25827711 10.1007/s40618-015-0269-8

[2] Hardefeldt PJ Eslick GD Edirimanne S. Benign thyroid disease is associated with breast cancer: a meta-analysis. Breast Cancer Res Treat. 2012;133:1169–77. 22434524 10.1007/s10549-012-2019-3

[3] Dobrinja C Scomersi S Giudici F. Association between benign thyroid disease and breast cancer: a single center experience. BMC Endocr Disord. 2019;19:104. 31623603 10.1186/s12902-019-0426-8 PMC6798360

[4] Mason C Yang R Hamilton R. Diagnosis of sarcoidosis from a biopsy of a dilated mammary duct. Proc (Bayl Univ Med Cent). 2017;30:197–9. 28405081 10.1080/08998280.2017.11929584 PMC5349827

[5] Goulabchand R Hafidi A Millet I. Mastitis associated with Sjögren’s syndrome: a series of nine cases. Immunol Res. 2016;65:218–29. 10.1007/s12026-016-8830-x 27561784

[6] Voizard B Lalonde L Sanchez LM. Lupus mastitis as a first manifestation of systemic disease: about two cases with a review of the literature. Eur J Radiol. 2017;92:124–31. 28624010 10.1016/j.ejrad.2017.04.023

[7] Goulabchand R Hafidi A Van de Perre P. Mastitis in autoimmune diseases: review of the literature, diagnostic pathway, and pathophysiological key players. J Clin Med. 2020;9:958. 32235676 10.3390/jcm9040958 PMC7231219

[8] Choi SH Jang KS Chung MS. Bilateral granulomatous mastitis with a different etiology. Cancer Biomark. 2015;15:333–8. 25519013 10.3233/CBM-140447 PMC12964679

[9] Kawataka M Kido T Tsuda R. A case of granulomatosis with polyangiitis with various breast lesions as the initial symptoms: a case-based review. Case Rep Rheumatol. 2021;2021:4416072. 34545315 10.1155/2021/4416072 PMC8448995

[10] Chougule A Bal A Das A Singh G. IgG4 related sclerosing mastitis: expanding the morphological spectrum of IgG4 related diseases. Pathology. 2015;47:27–33. 25474510 10.1097/PAT.0000000000000187

[11] Ecker NKJ Möslein G Ecker KW. Continent ileostomy: short- and long-term outcomes of a forgotten procedure. BJS Open. 2021;5:zrab095. 34686880 10.1093/bjsopen/zrab095 PMC8536872

[12] Park SH Choi SJ Jung HK. Sclerosing lymphocytic lobulitis manifesting as suspicious microcalcifications with Hashimoto’s thyroiditis in a young woman. Breast J. 2013;19:539–41. 23834442 10.1111/tbj.12156

[13] Onalan E Aslan M. Could neutrophil to lymphocyte ratio be a marker in Hashimoto’s thyroiditis? J Pak Med Assoc. 2020;70:1381–3. 32794490 10.5455/JPMA.32518

[14] Jiao Y Chang K Jiang Y Zhang J. Identification of periductal mastitis and granulomatous lobular mastitis: a literature review. Ann Transl Med. 2023;11:158. 36846004 10.21037/atm-22-6473 PMC9951018

[15] Ramadan R Koryem IM Fayed H. Idiopathic granulomatous mastitis: risk factors and management. Breast Dis. 2022;41:413–20. 36530069 10.3233/BD-220047

[16] Altintoprak F Kivilcim T Ozkan OV. Aetiology of idiopathic granulomatous mastitis. World J Clin Cases. 2014;2:852–8. 25516860 10.12998/wjcc.v2.i12.852 PMC4266833

[17] Şener Bahçe Z Aktaş H. Patients with idiopathic granulomatous mastitis accompanied by erythema nodosum. Int J Clin Pract. 2021;75:e13928. 33305438 10.1111/ijcp.13928

[18] Co M Cheng VCC Wei J. Idiopathic granulomatous mastitis: a 1-year study from a multicentre clinical database. Pathology. 2018;50:742–7. 30389215 10.1016/j.pathol.2018.08.010

[19] Sawicka-Gutaj N Gutaj P Sowiński J. Influence of cigarette smoking on thyroid gland--an update. Endokrynol Pol. 2014;65:54–62. 24549603 10.5603/EP.2014.0008

[20] Babić Leko M Gunjača I Pleić N Zemunik T. Environmental factors affecting thyroid-stimulating hormone and thyroid hormone levels. Int J Mol Sci. 2021;22:6521. 34204586 10.3390/ijms22126521 PMC8234807

[21] Gontarz-Nowak K Szychlińska M Matuszewski W Stefanowicz-Rutkowska M Bandurska-Stankiewicz E. Current knowledge on graves’ orbitopathy. J Clin Med. 2020;10:16. 33374706 10.3390/jcm10010016 PMC7793490

[22] Campos GC Castro MV de Mattos VF Pinto LZ Boechat MC Dos Santos AA. Lymphocytic mastopathy mimicking breast malignancy: a case report. Radiol Bras. 2014;47:256–8. 25741094 10.1590/0100-3984.2013.1847 PMC4337124

[23] Ragusa F Fallahi P Elia G. Hashimoto’s thyroiditis: epidemiology, pathogenesis, clinic and therapy. Best Pract Res Clin Endocrinol Metab. 2019;33:101367. 31812326 10.1016/j.beem.2019.101367

[24] Pyzik A Grywalska E Matyjaszek-Matuszek B Roliński J. Immune disorders in Hashimoto’s thyroiditis: what do we know so far? J Immunol Res. 2015;2015:979167. 26000316 10.1155/2015/979167 PMC4426893

[25] Aktas G Sit M Dikbas O. Elevated neutrophil-to-lymphocyte ratio in the diagnosis of Hashimoto’s thyroiditis. Rev Assoc Med Bras (1992). 2017;63:1065–8. 29489971 10.1590/1806-9282.63.12.1065

[26] Bilge M Yesilova A Adas M Helvaci A. Neutrophil- and platelet- to lymphocyte ratio in patients with euthyroid hashimoto’s thyroiditis. Exp Clin Endocrinol Diabetes. 2019;127:545–9. 30267388 10.1055/a-0723-3441

[27] Onalan E Dönder E. Neutrophil and platelet to lymphocyte ratio in patients with hypothyroid Hashimoto’s thyroiditis. Acta Biomed. 2020;91:310–4. 32420966 10.23750/abm.v91i2.8592 PMC7569628

[28] Erge E Kiziltunc C Balci SB. A novel inflammatory marker for the diagnosis of hashimoto’s thyroiditis: platelet-count-to-lymphocyte-count ratio. Diseases. 2023;11:15. 36810529 10.3390/diseases11010015 PMC9944872

[29] Xu J Huang G Weng L. Low serum interleukin-38 levels in patients with Graves’ disease and Hashimoto’s thyroiditis. J Clin Lab Anal. 2022;36:e24101. 34799942 10.1002/jcla.24101 PMC8761401

[30] Velidedeoglu M Kundaktepe BP Aksan H Uzun H. Preoperative fibrinogen and hematological indexes in the differential diagnosis of idiopathic granulomatous mastitis and breast cancer. Medicina (Kaunas). 2021;57:698. 34356979 10.3390/medicina57070698 PMC8303264

[31] Çetinkaya OA Çelik SU Terzioğlu SG Eroğlu A. The predictive value of the neutrophil-to-lymphocyte and platelet-to-lymphocyte ratio in patients with recurrent idiopathic granulomatous mastitis. Eur J Breast Health. 2020;16:61–5. 31912016 10.5152/ejbh.2019.5187 PMC6939709

[32] Li Q Wan J Feng Z Shi J Wei W. Predictive significance of the preoperative neutrophil-lymphocyte ratio for recurrence in idiopathic granulomatous mastitis patients. Am Surg. 2023;89:5577–83. 36880848 10.1177/00031348231161793

[33] Ciftci AB Bük OF Yemez K Polat S Yazicioğlu IM. Risk factors and the role of the albumin-to-globulin ratio in predicting recurrence among patients with idiopathic granulomatous mastitis. J Inflamm Res. 2022;15:5401–12. 36158516 10.2147/JIR.S377804 PMC9499730

[34] Chen W Wang J Ye B Zhou J Wang W. The population characteristics of the main leukocyte subsets and their association with chronic diseases in a community-dwelling population: a cross-sectional study. Prim Health Care Res Dev. 2021;22:e18. 33958026 10.1017/S1463423621000153 PMC8165331

[35] Qi X. Peripheral blood lymphocyte-to-monocyte ratio predicts mortality in patients with hbv-related decompensated cirrhosis. Clin Lab. 2019;65:1. 10.7754/Clin.Lab.2018.180717 30775885

